# Occupational performance characteristics in patients with bipolar disorders: discriminant analysis with major depressive disorders

**DOI:** 10.3389/fpsyt.2026.1844682

**Published:** 2026-07-29

**Authors:** Tomonari Hayasaka, Izumi Nagashima, Miku Hoshino, Koji Teruya, Yasuyuki Matumoto, Masami Murao, Taku Maruki, Masako Watanabe, Takeshi Katagiri, Mariko Kurihara, Yuki Oe, Yoshikazu Takaesu, Takashi Tsuboi, Koichiro Watanabe, Hitoshi Sakurai

**Affiliations:** 1 Department of Rehabilitation, Kyorin University School of Health Sciences, Tokyo, Japan; 2 Department of Neuropsychiatry, Kyorin University School of Medicine, Tokyo, Japan; 3 Department of Neuropsychiatry, Kyorin University Hospital, Tokyo, Japan; 4 Kyorin University School of Health Sciences, Tokyo, Japan; 5 Department of Neuropsychiatry, Graduate School of Medicine, University of the Ryukyus, Okinawa, Japan

**Keywords:** artistic activity, bipolar disorder, discrimination analysis, major depressive disorder, occupational therapy

## Abstract

**Introduction:**

This study aimed to examine whether occupational performance characteristics observed during occupational therapy could facilitate the differential diagnosis of individuals with bipolar disorder (BD) and major depressive disorder (MDD).

**Methods:**

A retrospective chart review was conducted on patients admitted to a university hospital for a one-week diagnostic evaluation. Eligible participants were aged 20–70 years, diagnosed with BD or MDD, and presented with at least mild depressive symptoms (Montgomery-Åsberg Depression Rating Scale ≥7). Behavioral data were systematically recorded during a structured occupational therapy session involving artistic activities. Occupational performance characteristics were identified via qualitative content analysis and coded as binary variables. Group differences were examined using nonparametric tests, followed by simultaneous discriminant analysis and receiver operating characteristic (ROC) analysis to evaluate diagnostic utility. In addition, leave-one-out cross-validation (LOOCV) was performed as an internal validation procedure to assess the stability of the discriminant model.

**Results:**

The study sample comprised 21 patients with BD and 35 patients with MDD. Among 66 identified occupational performance characteristics, 3 specific behaviors, namely “Messy work,” “Psychomotor dysregulation,” and “Work on your own without using a textbook,” were significantly more prevalent in the BD group. The discriminant model incorporating these characteristics achieved an overall classification accuracy of 80.4%, which was also observed in LOOCV. ROC analysis indicated moderate discriminative power (AUC = 0.765), characterized by relatively high specificity (94.3%) and moderate sensitivity (57.1%).

**Conclusions:**

Distinct occupational performance characteristics observed during occupational therapy provide clinically meaningful insights for differentiating BD from MDD during depressive episodes. Systematic behavioral observation in ecologically valid settings may serve as a valuable adjunct to conventional symptom-based diagnostic frameworks.

## 1 Introduction

Bipolar disorder (BD) and major depressive disorder (MDD) are prevalent mood disorders that impose a substantial burden on individuals and healthcare systems worldwide (1–3). BD is characterized by recurrent mood episodes, including depressive, manic, or hypomanic states, whereas MDD involves persistent depressed mood accompanied by anhedonia and cognitive and psychomotor disturbances (4, 5). Despite these distinctions, depressive episodes occur in both conditions, and differentiating bipolar depression from unipolar depression remains a major clinical challenge, particularly during depressive phases (6–9).

From a behavioral perspective, both BD and MDD are associated with impairments in daily functioning (10, 11). However, evidence suggests that behavioral patterns differ between the disorders. Individuals with BD often demonstrate greater behavioral variability, impulsivity, and instability in goal-directed activity, whereas those with MDD more typically exhibit behavioral inhibition and psychomotor slowing (12, 13). These differences are clinically relevant because they relate to illness course and functional outcomes. Differentiating BD from MDD is further complicated by the reliance on self-reported symptoms in routine clinical practice. During depressive states, impaired insight, cognitive distortions, and recall bias may reduce the reliability of retrospective symptom reporting (7, 14). This limitation is particularly relevant in BD, where past hypomanic episodes are frequently underrecognized, leading to misdiagnosis as MDD (15, 16). Recent discussions have highlighted that some patients initially diagnosed with treatment-resistant unipolar depression may later be recognized as having bipolar disorder, emphasizing the clinical importance of identifying subtle behavioral indicators associated with BD (17). Furthermore, patients may exaggerate improvements or conceal socially undesirable behaviors, and interview-based observations may not reflect daily functioning due to the restrictive clinical setting. Therefore, objective assessment in everyday-like contexts may be more useful for differentiating BD from MDD (18, 19).

Occupational therapy provides a valuable context for examining behavior during structured activities (20). Within this setting, clinicians can observe how mood-related symptoms are expressed in task performance, including task initiation, persistence, pacing, and interpersonal behavior. Such observation enables assessment of behavioral characteristics that may be difficult to capture through symptom rating scales or self-reported measures (21). Direct observation of occupational performance may therefore help distinguish behavioral manifestations of BD and MDD. Previous studies have reported differences in occupational engagement among individuals with mood disorders (22, 23). However, few studies have examined whether behavioral characteristics observed during occupational therapy can differentiate individuals with BD from those with MDD, particularly during depressive phases. Direct comparisons based on observable behavior within therapeutic activity contexts therefore remain limited.

Therefore, the present study aimed to compare behavioral and occupational performance characteristics observed during occupational therapy between individuals with BD and those with MDD. By focusing on observable patterns of task engagement rather than relying solely on self-reported symptoms, this study sought to clarify disorder-specific behavioral profiles that reflect underlying mood pathology in real-world activity settings.

## 2 Materials and methods

### 2.1 Study design

This retrospective study used clinical data from patients admitted to Kyorin University Hospital (Tokyo, Japan) for evaluation of difficult-to-treat depression between January 2015 and March 2019. Difficult-to-treat depression was defined as persistent depressive symptoms with insufficient response to standard treatments. Evaluation was conducted during a structured one-week hospitalization program for diagnostic clarification and treatment planning. Patients underwent standardized mental function assessments, and behavioral observations during occupational therapy sessions were recorded and retrospectively analyzed. Occupational performance characteristics observed during the sessions were subsequently screened for their potential utility in differentiating BD from MDD. All eligible patients were informed of the study and allowed to opt out of secondary data use. The study was approved by the Research Ethics Committee of the Faculty of Medicine, Kyorin University (approval number: R01-004). Details of the assessment program have been reported elsewhere (22, 23).

### 2.2 Participants

Participants were included if they: (a) underwent a one-week diagnostic hospitalization for comprehensive assessment of difficult-to-treat depression; (b) were aged 20–70 years; (c) were diagnosed with MDD or BD according to the Diagnostic and Statistical Manual of Mental Disorders, Fifth Edition (DSM-5); and (d) had clinically significant depressive symptoms, defined as a Montgomery-Åsberg Depression Rating Scale (MADRS) score ≥7 at admission. Exclusion criteria were: (a) current alcohol or substance use disorder; (b) imminent suicide risk requiring urgent intervention; (c) severe physical illness interfering with participation; or (d) clinically significant manic symptoms, defined as a Young Mania Rating Scale (YMRS) score ≥12 (24).

### 2.3 Occupational therapy program description

Occupational performance was assessed during a single structured occupational therapy session involving an artistic task (22, 23). Participants attended once and were informed in advance that the session was for observational assessment rather than therapeutic intervention. All sessions followed a predefined protocol including task explanation, therapist interaction, and behavioral recording (**Table 1**) to ensure methodological consistency. Participants selected one activity from several creative options (e.g., coloring, origami, knitting, leather crafting) with varying complexity.

**Table 1:** Implementation of artistic activity program.

Procedure	Details
a) Start of the program	Promotion of motivation for participation Confirmation of participation
b) Description of the program	Confirmation of content and purpose of the session
c) Icebreaker	Self-introduction of each participant
d) Selection of artwork task	Free selection of tasks from more than 10 choices with different levels
e) Implementation of the artwork task	Preparation of tools and materials Creation of the selected
f) End of the program	Present impressions for the session Cleaning up

The program was conducted in group sessions of approximately 10 participants.

### 2.4 Occupational performance characteristics evaluation

During the sessions, occupational therapists observed participants’ communicative behaviors, task engagement, and responses to environmental demands. After each session, therapists independently recorded observations of occupational performance characteristics before data integration. The data were analyzed using qualitative content analysis by three occupational therapists through collaborative review. The procedure included (1): extracting descriptions related to occupational performance from the records; (2) removing content unrelated to behavioral performance to generate raw data; (3) condensing the data while preserving essential meaning; and (4) organizing the condensed data according to semantic similarity and synthesizing them into categories representing occupational performance characteristics. Occupational performance characteristics were dichotomously coded as present or absent. The raters were blinded to the participants’ diagnostic status. In cases of discrepancy, consensus was reached through discussion among the therapists to determine the final classification.

### 2.5 Statistical analysis

Demographic and clinical variables, including age, sex, educational attainment, illness duration, and scores on the Montgomery-Åsberg Depression Rating Scale (MADRS), Young Mania Rating Scale (YMRS), Adult ADHD Self-Report Scale (ASRS), and Autism-Spectrum Quotient (AQ) as well as medication status (mood stabilizers, sedative or anxiolytic agents, antipsychotics, antidepressants), were obtained from medical records together with occupational performance characteristics. Group differences between the BD and MDD groups were examined using nonparametric tests. Continuous variables were analyzed with the Mann-Whitney U test, and categorical variables with the χ² test or Fisher’s exact test. Occupational performance characteristics showing significant group differences in the chi-square analysis (p < 0.05) were selected as candidate variables and simultaneously entered into the discriminant analysis model. Discriminative accuracy was evaluated using Receiver Operating Characteristic (ROC) curves and the Area Under the Curve (AUC), interpreted as high (>0.9), moderate (0.7-0.9), or low (0.5-0.7) (25). To further assess the stability of the discriminant model, leave-one-out cross-validation (LOOCV) was applied to the final discriminant model as an internal validation procedure after variable selection had been performed on the full dataset. In cases where occupational performance characteristics demonstrated low-frequency distributions, sensitivity analyses excluding those variables were additionally conducted to evaluate the robustness of the discriminant model. Analyses were conducted using SPSS for Windows, version 28, with statistical significance set at p < 0.05 (two-tailed). To assess inter-rater reliability, a random subset of cases was independently re-evaluated by two occupational therapists blinded to diagnostic information. Cohen’s κ coefficients were calculated for occupational performance characteristics selected for inclusion in the discriminant analysis model.

## 3 Results

### 3.1 Baseline demographics

The analytic sample comprised 21 patients with BD (11 males; mean age, 39.1 ± 11.4 years) and 35 patients with MDD (14 males; mean age, 41.1 ± 12.7 years). Comparisons of baseline demographic and clinical variables, and medication status between the BD and MDD patient groups demonstrated no statistically significant differences across any of the assessed variables (**Table 2**).

**Table 2:** Comparison of demographic characteristics, clinical variables, and medication status between the BD group and MDD group.

Variables	BD group (n=21)	MDD group (n=35)	*P* value
Age (years)	39.1 ± 11.4	41.1 ± 12.7	0.629
Male	11 (52.4%)	14 (40.0%)	0.415
University degree	9 (42.9%)	18 (51.4%)	0.582
Disease duration (years)	8.6 ± 5.5	9.7 ± 8.0	0.859
MADRS scores	21.6 ± 9.6	21.2 ± 8.0	0.852
YMRS scores	2.8 ± 3.1	1.4 ± 2.6	0.104
ASRS scores	2.4 ± 1.6	2.8 ± 1.8	0.458
AQ scores	25.3 ± 8.1	22.8 ± 6.7	0.244
Number of applicable occupational performance characteristics	10.4 ± 3.5	10.8 ± 3.7	0.740
Mood stabilizers (including lithium)	6 (28.6%)	5 (14.3%)	0.298
Sedative or anxiolytic agents	7 (33.3%)	10 (28.6%)	0.769
Antipsychotics	4 (19.0%)	4 (11.4%)	0.456
Antidepressants	3 (14.3%)	6 (17.1%)	1.000

BD, bipolar disorder; MDD, major depressive disorder.

MADRS, Montgomery Åsberg Depression Rating Scale; YMRS, Young Mania Rating Scale; ASRS, Adult ADHD Self-Report Scale; AQ, Autism Spectrum Quotient.

### 3.2 Comparison of each occupational performance characteristic

Across both diagnostic groups, a total of 66 distinct occupational performance characteristics were extracted, and their corresponding operational definitions based on observable behaviors are presented in **Supplementary Table 1**. Comparative analyses identified three characteristics that differed significantly between the BD and MDD groups (**Table 3**). Specifically, the BD group demonstrated a higher prevalence of the following behaviors: “Messy work,” “Psychomotor dysregulation,” and “Work on your own without using a textbook,” than the MDD group.

**Table 3:** Comparison of each occupational performance characteristics between the BD group and MDD group.

Variables	BD group (n=21)	MDD group (n=35)	*P* value
Messy work	8 (38.1%)	1 (2.9%)	< 0.001 **
Work on your own without using a textbook	5 (23.8%)	1 (2.9%)	0.024 *
Psychomotor dysregulation	3 (14.3%)	0 (0.0%)	0.048 *

BD, bipolar disorder; MDD, major depressive disorder.

* p < 0.05, ** p < 0.01.

### 3.3 Discriminant analysis by occupational performance characteristics

**Figure 1:**
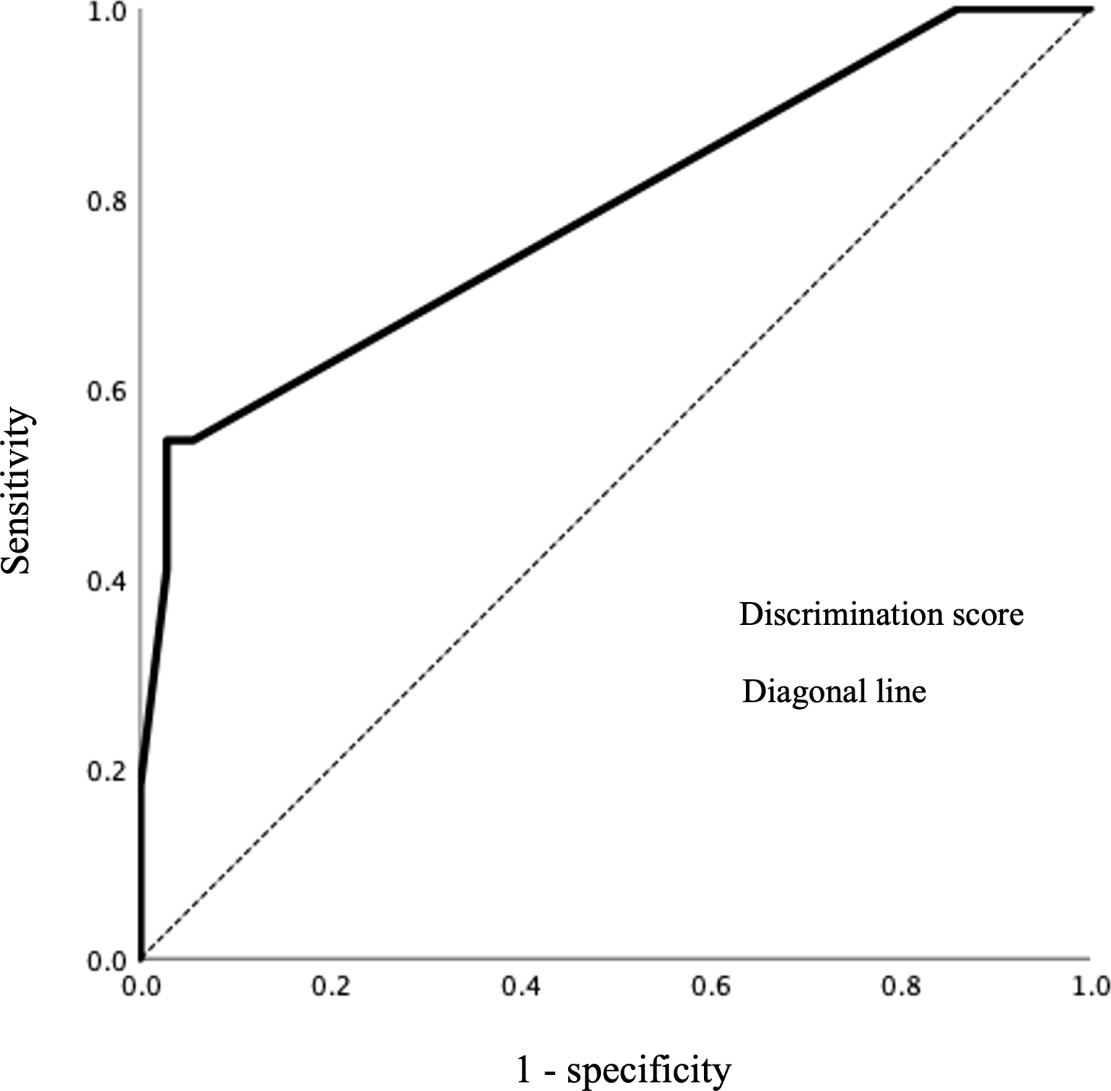
Receiver operating characteristic curves of discriminant score.

Simultaneous discriminant analysis was performed to distinguish between the BD and MDD groups, using group membership as the dependent variable and the three occupational performance characteristics identified in the preceding analyses as independent variables. This analysis yielded a linear discriminant function expressed as follows: 2.294 × “Messy work” + 2.071 × “Psychomotor dysregulation” + 1.510 × “Work on your own without using a textbook” - 0.641. The model demonstrated a Wilks’ λ of 0.671 (p < 0.001), with an overall correct classification rate of 80.4% (**Table 4**). To further evaluate the discriminative performance of the model, a Receiver Operating Characteristic (ROC) analysis was conducted using the discriminant scores derived from the linear function. The resulting AUC was 0.765 (95% CI = 0.622-0.908, p < 0.0001), indicating moderate classification accuracy. At the optimal cutoff value of 0.114, sensitivity was 57.1%, and specificity was 94.3% (**Figure 1**). To assess the robustness of the model, LOOCV was performed, yielding the same overall classification accuracy of 80.4%, thereby suggesting relative stability of the discriminant model. In addition, because “Psychomotor dysregulation” demonstrated a low cell frequency, a sensitivity analysis excluding this variable was conducted. The reduced two-variable model yielded an AUC of 0.738 (95% CI = 0.591–0.885, p < 0.001), sensitivity of 52.4%, and specificity of 94.3%, indicating that the overall discriminative performance was largely preserved. *Post hoc* inter-rater reliability analyses demonstrated substantial agreement for the occupational performance characteristics included in the discriminant analysis model. Cohen’s κ coefficients were 0.875 for “Messy work,” 0.643 for “Psychomotor dysregulation,” and 0.773 for “Work on your own without using a textbook”.

**Table 4:** Discriminant analysis by occupational performance characteristics.

Analytic factors	Standardized canonical discriminant function coefficient	Canonical discriminant function coefficient	Wilks’ λ p value
Messy work	0.760	2.294	< 0.001 **
Work on your own without using a textbook	0.452	1.510	0.014 *
Psychomotor dysregulation	0.449	2.071	0.021 *

These variables produced a linear discriminant formula: 2.294 × “Messy work” + 2.071 × “Psychomotor dysregulation” + 1.510 × “Work on your own without using a textbook” − 0.641.

The linear discriminant function was developed by calculating discriminant scores from the data obtained using the binary classification method of the occupational performance characteristic.

P values were derived from tests of equality of group means in the discriminant analysis.

* p < 0.05, ** p < 0.0.1.

## 4 Discussion

To our knowledge, this study is the first to examine behavioral characteristics observed during occupational therapy that differentiate patients with BD from those with MDD using discriminant analysis. Three occupational performance characteristics “Messy work,” “Psychomotor dysregulation,” and “Work on your own without using a textbook” were identified as significant discriminators between the groups. These variables were incorporated into a linear discriminant function: 2.294 × “Messy work” + 2.071 × “Psychomotor dysregulation” + 1.510 × “Work on your own without using a textbook” − 0.618, yielding a discrimination accuracy of 80.4%. The model showed relatively high specificity, suggesting that these behavioral characteristics may help identify patterns unlikely to reflect MDD alone. In addition, a sensitivity analysis excluding the low-frequency variable “Psychomotor dysregulation” demonstrated comparable discriminative performance, supporting the robustness of the model. *Post hoc* inter-rater reliability analyses demonstrated substantial agreement for the occupational performance characteristics included in the discriminant model, supporting the reproducibility of the behavioral evaluations. Overall, the findings indicate that observable occupational performance characteristics may provide clinically meaningful information for differentiating BD from MDD, particularly when symptomatic overlap complicates diagnosis, consistent with recent perspectives emphasizing the risk of bipolar misdiagnosis in treatment-resistant depression cohorts (17). Therefore, the present findings should be interpreted cautiously as exploratory and hypothesis-generating until validated in independent cohorts.

Each of the three occupational performance characteristics is consistent with established features of BD. The characteristic “Messy work” identified in this study likely reflects the complex interaction of cognitive functioning, mood regulation, and personality-related tendencies associated with BD (26). Even during depressive phases, individuals with BD often exhibit instability in attentional allocation (27), information integration (28), and executive organization (29), which can adversely affect the ability to maintain coherence and structure throughout task performance. Fluctuations in mood and mental energy may further compromise sustained concentration and self-monitoring (30), leading to a prioritization of immediacy or subjective momentum over precision and orderly execution. In addition, heightened emotional reactivity and impulsive cognitive styles (30, 31), which are frequently reported in BD, may reduce sensitivity to task aesthetics or consistency, resulting in disorganized or uneven output. From this perspective, this characteristic may reflect cognitive–emotional instability during goal-directed activities in individuals with BD.

In contrast, the characteristic “Psychomotor dysregulation” is more appropriately understood as reflecting difficulties in behavioral inhibition, motor regulation, and self-control, rather than impaired fine motor ability per se. In the present study, this behavior was observed more frequently in patients with BD, indicating potential instability in the regulation of force, speed, and precision during task execution. Individuals with BD may experience residual inner tension, heightened arousal, or psychomotor dysregulation even outside overt manic states, which can manifest as excessive force or rigidity during manual tasks (32, 33). Such overactivation may interfere with the smooth modulation of movement, resulting in reduced precision despite intact motor capacity (31). Furthermore, impairments in self-monitoring and behavioral adjustment may limit the individual’s ability to recognize and correct overly forceful or poorly controlled movements once initiated (34, 35). Accordingly, “Psychomotor dysregulation” could be conceptualized not merely as a motor skill deficit, but as a possible behavioral manifestation of insufficient inhibitory control and unstable action regulation.

The characteristic “Work on your own without using a textbook” may reflect reduced reliance on external task frameworks or shared procedural references (36). In occupational therapy settings, reference materials typically support task structuring, error monitoring, and alignment with expected procedures. A tendency to proceed without such support may indicate diminished awareness of task demands, impulsive engagement, or subtle disinhibition (31, 37). In patients with BD, these behaviors may be observed even in the absence of overt manic symptoms and may reflect underlying vulnerabilities in cognitive control and self-monitoring (28, 38). Importantly, such behavior does not necessarily represent intentional independence; rather, patients may initiate and continue tasks based on their own judgment without adequately incorporating external guidance, which can lead to inefficient task execution or failure to achieve intended goals and, in turn, may contribute to subsequent depressive states (39).

The discriminant function showed moderate accuracy with relatively high specificity, suggesting that occupational performance characteristics may help reduce diagnostic ambiguity between BD and MDD rather than serve as a broad screening tool (8). Misdiagnosis of BD as MDD remains a major clinical problem affecting treatment selection and prognosis (12, 15). The present findings indicate that structured observation of occupational performance may improve diagnostic formulation by capturing behavioral instability that is not easily detected through self-report or brief interviews (14, 40). Previous studies have suggested that peripheral biological markers have limited utility in accurately predicting cognitive and mood states (41). Occupational therapy offers an ecologically valid setting for such assessment, enabling real-time observation of behavior in a structured activity context (22, 23). Behaviors such as task organization, motor coordination, and the use of instructional materials may reveal clinically meaningful patterns that are not readily captured in conventional psychiatric evaluations (29, 35, 42). Given that psychiatric interventions play a critical role in reducing occupational dysfunction and improving quality of life (43), the findings of this study may contribute to more accurate diagnosis and the development of treatment plans informed by functional insights.

Several limitations should be considered. First, the sample size was relatively small and drawn from a single institution, which may limit the generalizability of the findings. In addition, the assessment was based on a single occupational therapy session involving artistic activities, potentially limiting the ecological and external validity of the observed behavioral patterns. Second, the BD group consisted of patients who initially presented with treatment-resistant or pseudo-resistant depression and were subsequently rediagnosed with BD during the one-week diagnostic program. Therefore, the discriminant model derived from this cohort should be interpreted cautiously and should not be generalized to first-episode or community samples without independent external validation. Third, although LOOCV was performed as an internal validation procedure to assess model stability, this approach does not constitute external validation, and the possibility of overfitting and limited generalizability cannot be fully excluded. In addition, variable selection was performed on the full dataset prior to LOOCV, and therefore the cross-validation procedure did not include repeated variable selection within each fold. Consequently, the reported classification performance may still reflect optimistic bias. Some occupational performance characteristics demonstrated low-frequency distributions, which may have affected the stability of the discriminant model despite the sensitivity analyses performed. Furthermore, no correction for multiple testing was applied during the initial univariate screening of occupational performance characteristics. Given the large number of comparisons performed, some statistically significant findings may have occurred by chance. Fourth, although *post hoc* inter-rater reliability analyses demonstrated substantial agreement for the occupational performance characteristics included in the discriminant model, reliability was assessed only in a subset of cases and was not evaluated across all behavioral variables or occupational therapy sessions. Fifth, although efforts were made to minimize bias through consideration of potential confounding factors, blinded assessments, and consensus discussions among multiple therapists, the retrospective design and therapist-rated assessments may still have introduced observer bias. Sixth, mood state at the time of assessment was not strictly controlled, and subthreshold mood fluctuations may have influenced participants’ behaviors. Furthermore, occupational performance was evaluated only during the artistic activity program and may not fully reflect behavior in daily life. Seventh, although age and sex were considered, other potentially relevant factors, such as occupational history and educational background, were not fully examined. Finally, although medication status was examined, its potential influence on the three identified behavioral characteristics could not be fully controlled, and residual confounding cannot be ruled out. Future prospective multi-center studies using standardized assessment protocols and external validation cohorts are warranted to confirm and extend the present findings.

## 5 Conclusion

In conclusion, specific occupational performance characteristics observed during occupational therapy may help differentiate BD from MDD. These behaviors appear to reflect underlying instability in behavioral regulation and cognitive control associated with BD, even outside overt manic states. Systematic observation of occupational performance in therapeutic settings may therefore complement symptom-based diagnostic approaches, particularly when depressive presentations overlap. Further multi-center studies are needed to validate these findings and examine their potential use in diagnostic support tools.

## Data Availability

The datasets presented in this study can be found in online repositories. The names of the repository/repositories and accession number(s) can be found in the article/**Supplementary Material**.
