# Supplementary material for: Occupational performance characteristics in patients with bipolar disorders: discriminant analysis with major depressive disorders
Source: Front Psychiatry. 2026 Jul 29;17:1844682. doi: 10.3389/fpsyt.2026.1844682 (PMC13461424; doi:10.3389/fpsyt.2026.1844682)
Supplement: Supplementary file 1 [file Table1.docx]

Supplementary Table 1. Occupational performance characteristics evaluated through an artistic activity program in occupational therapy: items and operational definitions

| **No.** | **OPC (No.1-17)** | **Operational Definition** |
| --- | --- | --- |
| 1 | Accept suggestions from others | Accept or incorporate suggestions from therapists or other participants, including imitating others’ approaches or work |
| 2 | Altruism | Prioritize helping others with their tasks or work, even at the expense of one’s own progress |
| 3 | Ask questions and consult | Appropriately ask questions or seek guidance when uncertain about tasks or procedures |
| 4 | Bring personal items into the OT room | Bring personally owned materials required for the activity into the therapy room |
| 5 | Calm as you get used to the place | Show increasing relaxation and more positive or softened facial expressions as familiarity develops |
| 6 | Careful and attentive in working | Engage in deliberate and cautious task execution with repeated adjustments and trial-and-error |
| 7 | Change activities frequently | Repeatedly shift between different tasks regardless of completion status |
| 8 | Choose or seek new activities | Select or request unfamiliar or more challenging tasks |
| 9 | Choose simple work | Select tasks with lower complexity or difficulty |
| 10 | Clean up or help clean up | Voluntarily participate in or initiate post-session cleanup |
| 11 | Clearly uplifting mood | Demonstrate a marked improvement in mood, reflected in observable affect or a two-level increase in self-rating |
| 12 | Concentrate on work | Sustain attention on the task with minimal distraction or interaction |
| 13 | Create work at a high degree of perfection | Produce work with high completeness, accuracy, and aesthetic quality |
| 14 | Difficulty in choosing activities | Show difficulty selecting tasks independently or require extended time or assistance |
| 15 | Difficulty in continuously participating in OT | Display interruptions in engagement, such as pausing, staring, or disengagement |
| 16 | Difficulty in finishing work | Continue working despite session completion cues or instructions to stop |
| 17 | Difficulty in handling some tools | Demonstrate inconsistent ability when handling different tools |

Supplementary Table 1. (continued)

| **No.** | **OPC (No.18-34)** | **Operational Definition** |
| --- | --- | --- |
| 18 | Difficulty in understanding procedures | Fail to understand instructions or require repeated explanations |
| 19 | Discard the work without any attachment | Express no attachment to completed work and decline to keep it |
| 20 | Dislike artistic activities | Verbally or non-verbally express aversion toward artistic tasks |
| 21 | Excessive confirmation | Repeatedly check instructions or ask the same questions multiple times |
| 22 | Excessive reactions to others | Exhibit exaggerated verbal or behavioral responses to others or environmental stimuli |
| 23 | Expressionless | Show minimal or no variation in facial expression during participation |
| 24 | Feel down and decrease self-esteem | Demonstrate a decline in mood or self-evaluation after the session |
| 25 | Feel uncomfortable in groups | Show reluctance or inability to participate in group settings |
| 26 | Fluent conversations | Engage in smooth and uninterrupted verbal communication |
| 27 | Frequent changes in emotions during activities | Display rapid and noticeable fluctuations in emotional expression |
| 28 | Give instructions to medical staff during activities | Direct therapists or staff regarding task-related actions |
| 29 | Have its own manners | Display idiosyncratic behavioral patterns or personal rules in interaction or task performance |
| 30 | Have negative remarks | Verbalize lack of ability or confidence (e.g., “I cannot”) |
| 31 | High self-esteem | Express strong confidence or assign high self-evaluation |
| 32 | Impaired body schema | Show difficulty imitating movements or exhibit inappropriate motor coordination |
| 33 | Lack of completion | Fail to complete tasks within the session time |
| 34 | Lapses in the process | Omit or skip necessary steps during task execution |

Supplementary Table 1. (continued)

| **No.** | **OPC (No.35-51)** | **Operational Definition** |
| --- | --- | --- |
| 35 | Lots of comments in OT note | Produce an unusually large amount of written self-report |
| 36 | Makeshift work | Engage superficially without sustained effort or seriousness |
| 37 | Messy hair or clothes | Display noticeable disorganization in personal appearance |
| 38 | Messy work | Produce disorganized or poorly structured work with low attention to detail |
| 39 | Misunderstand processes | Repeat errors in procedures despite correction or feedback |
| 40 | Nervous in interpersonal interactions | Exhibit signs of anxiety such as blushing, sweating, or hesitant speech |
| 41 | Never or rarely converse with others | Engage in little or no verbal interaction during the session |
| 42 | No eye contact | Avoid or fail to establish eye contact during interaction |
| 43 | Not seek help | Do not request assistance despite evident difficulty |
| 44 | Obvious fatigue | Show or report fatigue, sleepiness, or physical discomfort |
| 45 | Pleased to be praised | Demonstrate visible positive emotional responses to praise |
| 46 | Psychomotor Dysregulation | Use excessive or insufficient force or show irregular motor control during tasks |
| 47 | Punctual | Arrive before or at the scheduled start of the session |
| 48 | Refuse suggestions | Reject or do not accept suggestions from others |
| 49 | Repetition of the same behavior | Repeat actions unnecessarily despite adequate completion |
| 50 | Restless | Display excessive movement, frequent standing, or inability to remain seated |
| 51 | Roundabout way of conversation | Communicate in an indirect, lengthy, or unfocused manner |

Supplementary Table 1. (continued)

| **No.** | **OPC (No.52-66)** | **Operational Definition** |
| --- | --- | --- |
| 52 | Satisfaction with OT | Express or demonstrate satisfaction with the activity |
| 53 | Self-rating 2 levels up after OT | Show a two-level increase in self-evaluation after the session |
| 54 | Shy during conversations | Display hesitancy, embarrassment, or avoidance in communication |
| 55 | Soliloquize while working | Talk to oneself during task performance without delusional content |
| 56 | Start to talk to others once adapted | Initiate interaction after becoming familiar with the environment or activity |
| 57 | State that OT is fun | Verbally express enjoyment or show observable enthusiasm |
| 58 | Sudden interruption of work | Stop participation abruptly and do not resume |
| 59 | Superficial communication | Engage in repetitive or shallow responses lacking depth or emotional engagement |
| 60 | Take a break during the program | Request or take a rest due to discomfort or fatigue |
| 61 | Take a long time to be seated | Show delayed or hesitant seating behavior upon entering the room |
| 62 | Try to get others to do their work | Attempt to have others complete tasks on their behalf |
| 63 | Unclear conversation contents | Produce speech that is difficult to understand or lacks clarity |
| 64 | Work on your own without using a textbook | Perform tasks without referring to provided instructions or materials |
| 65 | Work only at your own pace | Work at a pace inconsistent with session expectations or structure |
| 66 | Work too far ahead | Proceed to subsequent steps prematurely without instruction |

OPC, occupational performance characteristic; OT, occupational therapy

The order of listing is A to Z.
